# Metagenome-assembled genome sequences of two cyanobacterial cultures from Homa Bay County, Kenya

**DOI:** 10.1128/mra.01205-23

**Published:** 2024-02-20

**Authors:** Katelyn M. Brown, Christopher S. Ward, George S. Bullerjahn

**Affiliations:** 1 Department of Biological Sciences, Bowling Green State University, Bowling Green, Ohio, USA; 2 Great Lakes Center for Fresh Waters and Human Health, Bowling Green State University, Bowling Green, Ohio, USA; University of Southern California, Los Angeles, California, USA

**Keywords:** cyanobacteria, microcystins, Winam Gulf, Lake Simbi

## Abstract

Metagenome-assembled genomes were generated for two xenic cyanobacterial strains collected from aquatic sources in Kenya and sequenced by NovaSeq S4. Here, we report the classification and genome statistics of *Microcystis panniformis* WG22 and *Limnospira fusiformis* LS22.

## ANNOUNCEMENT

*Microcystis* spp. are cosmopolitan cyanobacteria that tolerate a wide range of temperature conditions, blooming at a minimum of 15°C (1). *Arthrospira* (*Limnospira*) spp. are primarily found in Africa, Asia, South America, and Central America, and occur in soda lakes in the African Rift Valley (2). In June 2022, a *Microcystis* strain was collected from the Winam Gulf offshore of Homa Bay (−0.494583, 34.444167) using a plankton net. Also in June 2022, a *Limnospira* strain was collected in Homa Bay County from Lake Simbi (−0.367750, 34.629833) from the shoreline. Xenic cyanobacterial cultures were separated through a dilution series by selectively pipetting colonies/filaments from 10 µL of original sample on a microscope slide into 10 µL of BG-11 media, repeating until single colonies/filaments were achieved. The resulting *Microcystis* colony was transferred into fresh liquid BG-11 media (https://utex.org/products/bg-11-medium?variant=30991786868826#recipe) in a 24-well plate and incubated at 21°C and 5 µmol/m^2^/s until biomass accumulated. *Limnospira* was grown in liquid Spirulina media (pH 10.4; https://utex.org/products/spirulina-medium?variant=30991737454682#recipe). Unialgal growth was confirmed by microscopy, and biomass was transferred into 25 mL of fresh media in a culturing flask.

The xenic cyanobacterial cultures were cultured at the conditions stated above and monitored for growth. After several months of acclimation, approximately 20 mL of dense culture was filtered through a Sterivex filtration unit (0.22 µm pore size, Sigma Aldrich, St. Louis, MO). Filters were frozen until extraction, where the membranes were removed from the plastic casing, and DNA was extracted using a DNeasy PowerWater Kit (Qiagen, Germantown, MD) according to manufacturer’s instructions. Eluted DNA was sequenced at the University of Minnesota Genomics Center. Unique Dual Indexed Illumina DNA libraries were prepared using Nextera DNA Flex and sequenced on a NovaSeq S4 to generate 150-bp paired-end metagenomic reads. Paired-end reads were input into the Department of Energy Systems Biology Knowledgebase for *de novo* assembly of each metagenome-assembled genome (MAG) in separate workflows [KBase; (3)]. Default parameters were used for all applications listed unless otherwise specified. Reads were imported as a paired-end library and trimmed to eliminate low-quality base calls and Nextera-PE sequencing adapters, also setting the head crop length to 15 [Trimmomatic v0.36; (4)]. After trimming, read quality was assessed using FastQC (v0.11.9; https://www.bioinformatics.babraham.ac.uk/projects/fastqc/). Metagenomic reads were assembled using metaSPAdes [v3.15.3; (5)] and binned with MaxBin2 [v2.2.4; (6)], MetaBAT2 [v1.7; (7)], and CONCOCT [v1.1; (8)] with minimum contig length of 2,000 bp prior to bin optimization using DAS-Tool [v1.1.2; (9)]. Bin quality was assessed with CheckM [v1.0.18; Table 1; (10)], and the bins classified to cyanobacteria were extracted. The quality of binned assemblies was assessed using QUAST [v4.4; (11)], and taxonomy was assigned to MAGs with the Genome Taxonomy Database (GTDB-Tk v1.7.0) and FastANI (12–17). Finally, MAGs were annotated using the Prokaryotic Genome Annotation Pipeline (PGAP) through National Center for Biotechnology Information [v6.6; (18–20)]. Additional analysis using AntiSMASH v7.0 indicated that WG22 has a complete *mcy* operon that correlates with frequent detections of microcystins in the Winam Gulf, while LS22 is not predicted to produce any common cyanotoxins (21, 22).

**TABLE 1:** Summary of metagenome-assembled genomes WG22 and LS22

Characteristic	*Microcystis panniformis* WG22	*Limnospira fusiformis* LS22
Assembly GenBank accession no.	SAMN37196505	SAMN37196506
Raw reads GenBank accession no.	SAMN36615257	SAMN36615258
No. of reads	408,836,714	506,895,224
MAG length (bp)	4,264,909	5,209,155
Bin completeness (%)	92.12	98.18
Bin contamination (%)	3.51	0.44
No. of reference genomes for marker sets	79	79
No. of marker genes	582	584
No. of marker sets	456	458
Marker genes identified zero times	46	10
Marker genes identified one time	517	571
Marker genes identified two times	19	3
Average GC content (%)	42.71	44.5
No. of contigs	532	407
*N*_50_ (bp)	13,379	20,557
No. of predicted genes	4,170	4,801
FastANI reference	GCF_010196425.1	GCA_012516315.1
FastANI reference identity	*Microcystis panniformis*	*Limnospira fusiformis*
FastANI ANI (%)	95.6	99.38

## Data Availability

The metagenome-assembled genomes have been deposited in GenBank under the accession numbers JAVSPN000000000 and JAVSPO000000000 for WG22 and LS22, respectively. The versions described in this paper are versions JAVSPN010000000 and JAVSPO010000000. The raw sequence files are available as sequence read archives under SRR25339258 and SRR25339257. The BioProject can be found under the reference PRJNA996591.
